# Abdominal pseudohernia caused by thoracic disk herniation: case series and review of the literature

**DOI:** 10.1093/jscr/rjae822

**Published:** 2025-01-23

**Authors:** G Massé, M Al Khaldi, F Schwenter, M Boudier-Revéret, H Sebajang

**Affiliations:** Digestive Surgery Service, Department of Surgery, Centre Hospitalier de l’Université de Montréal (CHUM), 1000 rue St-Denis, Montreal, QC H2X 0C1, Canada; Digestive Surgery Service, Department of Surgery, Centre Hospitalier de l’Université de Montréal (CHUM), 1000 rue St-Denis, Montreal, QC H2X 0C1, Canada; Digestive Surgery Service, Department of Surgery, Centre Hospitalier de l’Université de Montréal (CHUM), 1000 rue St-Denis, Montreal, QC H2X 0C1, Canada; Department of Physical Medicine and Rehabilitation, Centre Hospitalier de l’Université de Montréal (CHUM), 1000 rue St-Denis, Montreal, QC H2X 0C1, Canada; Digestive Surgery Service, Department of Surgery, Centre Hospitalier de l’Université de Montréal (CHUM), 1000 rue St-Denis, Montreal, QC H2X 0C1, Canada

**Keywords:** abdominal wall bulge, abdominal wall hernia, intervertebral thoracic disk herniation, pseudohernia, intervertebral disk hernia

## Abstract

Intervertebral thoracic disk herniation (TDH) is a rare occurrence and presents with a wide variety of symptoms. Errors in diagnosis are thought to be frequent due to the variable clinical presentations. We herein present two unusual cases of TDH presenting with abdominal pseudohernias, abdominal pain, and hypoesthesia along the T11-T12 dermatomes due to TDH at the same level. Both patients were managed conservatively, using a combination of analgesics and muscle relaxants. At 10 months of follow-up, the first patient reported complete resolution of abdominal bulge and no residual pain. The second patient reported residual paresthesia of T11-T12 dermatome, with non-limiting back pain and almost complete resolution of abdominal bulge. In conclusion, TDH may present with an abdominal wall bulge mimicking hernia, hypoesthesia, and radicular pain along the affected dermatome. Conservative management can be considered as first-line treatment.

## Introduction

Intervertebral thoracic disk herniation (TDH) accounts for <1% of all intervertebral disk herniations [1, 2]. They are mostly seen in patients between 30 and 50 years old [3], with no significant difference between males and females [4]. The most common location lies between T7 and T12 [1, 2]. TDH leads to symptoms in <30% of cases. The symptoms are usually slowly progressive [5]. They include back pain, non-visceral chronic abdominal pain [6–9], and myelopathy. However, over the years, many physicians have described atypical presentations, such as dyspnea, thoracic wall pain, nausea, and abdominal-related symptoms [3, 7, 9–11]. Among the abdominal-related symptoms, alterations of bowel movements, tenesmus, and bladder dysfunction are the most frequent [7].

This wide array of presenting symptoms may lead to misdiagnosis [1]. Patients often undergo numerous investigations before any diagnosis is given [12]. It can take up to a year after onset of symptoms before the patient is referred to a spine specialist [9]. Delays in accurate diagnosis and management can lead to serious complications, especially neurological compromise. Recognition of atypical presentation of TDH is therefore important [4, 9].

In this article, we describe two cases of TDH initially misdiagnosed as flank hernias. The presenting symptoms, workup, and management are described.

## Case presentation

### Patient 1

A 52-year-old male with asthma, type 2 diabetes mellitus, and no surgical history presented to a general surgeon’s office for a painful left flank bulge, suggestive of a hernia. Further history taking revealed that the patient had previously visited the emergency department (ED) several weeks prior for back pain radiating to the umbilicus with an associated left flank bulge. The pain had started after exercising, and was described as a constant burning sensation radiating along the T11-12 dermatomes exacerbated with physical activity. After excluding signs of myelopathy and cauda equina syndrome at the ED, the patient was discharged with analgesia, muscle relaxants, and an outpatient consultation with general surgery for hernia investigation. A computed tomography (CT) scan, however, failed to reveal a hernia. There was no evidence of obstructive intestinal symptoms. The patient was referred to physical medicine and rehabilitation by the surgeon.

**Figure 1:**
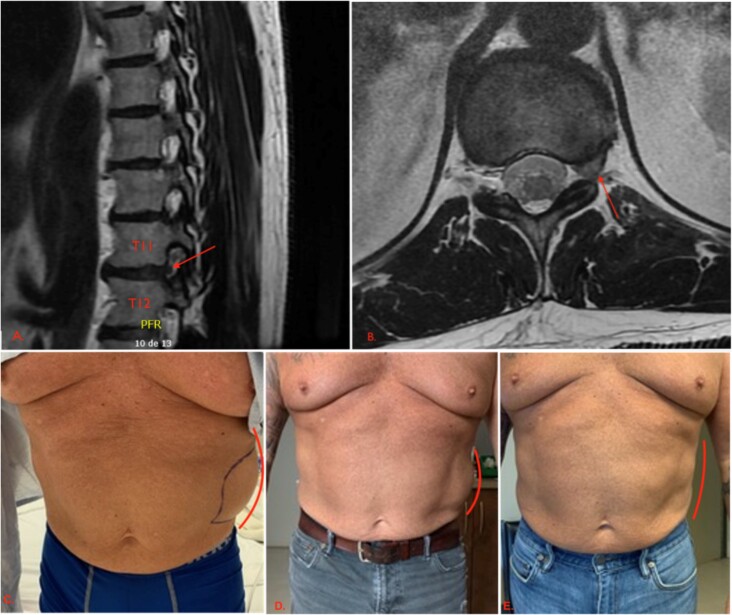
First patient’s initial presentation and clinical evolution. (A) An axial view of a CT scan of the abdomen and pelvis revealing a left-sided flank bulge consistent with the patient’s complaint without evidence of hernia. (B, C, D) Left flank bulge at, respectively, 1, 3, and 10 months follow-up, showing a progressive reduction in size (lines). The pictures were given with the patient’s consent.

A detailed physical exam conducted by the physiatrist revealed hypoesthesia along the left T11-T12 dermatomes without tenderness around the corresponding paravertebral space, and no motor or sensory deficits in the extremities. Subsequent magnetic resonance imaging (MRI) of the dorsolumbar spine revealed a small left-sided foraminal disc herniation at the T11-T12 levels causing stenosis of the left foramen, consistent with the patient’s symptoms (Fig. 1). The patient was treated conservatively with gabapentin and presented significant improvement of symptoms 3 months following the initiation of treatment, with complete resolution of both symptoms and abdominal bulge at 10-month follow-up.

### Patient 2

**Figure 2:**
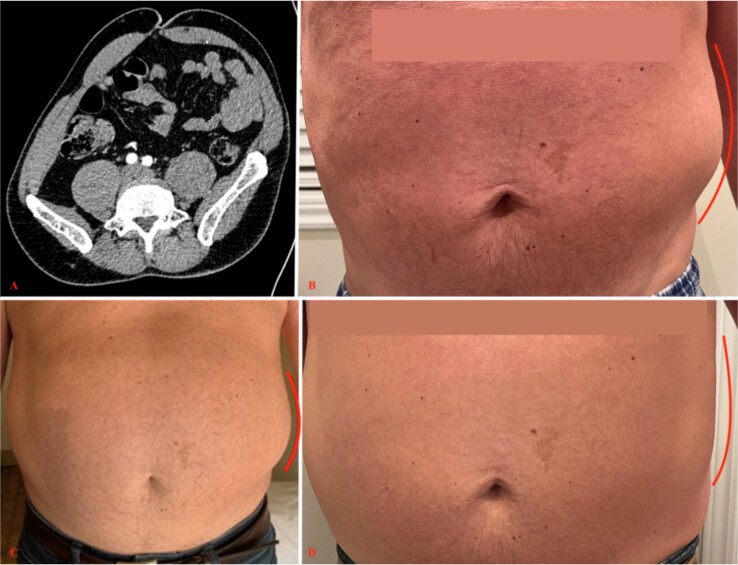
Second patient’s initial scan and clinical evolution. (A) An axial view of a CT scan of the abdomen and pelvis revealing a left-sided flank bulge consistent with the patient’s complaint without evidence of hernia. (B, C, D) Left flank bulge at, respectively, 1, 3, and 10 months follow-up, showing progressive reduction in size (lines). The pictures were given with the patient’s consent.

A 57-year-old male with a medical history of recurrent renal colic and cervical disk hernia presented to the ED with a left flank bulge and associated pain (Fig. 2). The patient reported experiencing a sudden sharp pain in the left back, radiating down to the groin, after lifting a heavy object, along with a left flank bulge. Physical examination revealed the presence of allodynia and hypoesthesia along the left T11-T12 dermatomes.

Given the initial suspicion of an abdominal hernia, a CT scan of the abdomen and pelvis was performed, but no significant findings, including hernias and renal stones, were detected. A left-sided deviation of the abdomen was noted (Fig. 2).

An MRI of the dorsolumbar spine was considered, but the patient’s severe pain precluded the possibility of remaining still for the duration of the examination. As an alternative, a CT scan of the dorsolumbar spine was conducted, revealing a small, herniated disk extending over 6 mm in the region of the left foramen of T11-T12. The patient was managed conservatively by the same physiatrist. The patient’s symptoms showed significant improvement at the 3-month follow-up visit, but persistent paresthesia on T11–12 dermatome and residual non limiting back pain were reported at the 10-month follow-up (Fig. 2).

## Discussion

In this report, we described two patients with intervertebral thoracic disk hernias at the T11-T12 levels. The first patient, a 52-year-old man, presented with left abdominal bulge, abdominal pain, and hypoesthesia along the dermatome. The second patient, a 57-year-old man, presented with left abdominal wall bulge, pain radiating to the groin, and hypoesthesia along the T11-T12 dermatome. Both underwent abdominal CT scans, which allowed the exclusion of abdominal wall hernias.

The diagnostic spectrum of abdominal wall bulge encompasses a broad range of conditions. Abdominal malignancies, abdominal wall hernias and recent abdominal or spine surgeries must be excluded before considering a pseudohernia [3, 13, 14]. A pseudohernia is a bulge in the abdominal wall, resembling a true hernia, but without fascial defect. Pseudohernias are usually the result of an underlying segmental neuropathy with subsequent abdominal wall musculature denervation. Diabetic thoracic radiculopathy, herpes zoster radiculopathy, paralytic poliomyelitis [14], syringomyelia [15], rib fracture [16], and intervertebral disk herniation should be considered.

In both cases presented, a thorough review of the medical history did not reveal any indicators of viral infections. Although the first patient was undergoing treatment for type 2 diabetes mellitus, there were no apparent indications of classic diabetic neuropathy, making the diagnosis of diabetic thoracic radiculopathy less likely. However, in the event that the dorsolumbar imaging had not revealed intervertebral disk hernias in both cases, electromyography could have been considered as an additional diagnostic tool [17].

The dorsolumbar CT scan of the second patient revealed the presence of several disc microcalcifications at multiple levels, specifically in the T12-L1 and T10-T11 disks. These findings raised the possibility of microcrystalline arthropathy. Nevertheless, it is important to note that no inflammatory signs were observed on the CT scan, and C-reactive protein levels were within the normal range.

Abdominal pain, regardless of the presence of abdominal wall bulging, is frequently attributed to intra-abdominal visceral causes, leading to the initiation of various investigations. Consequently, many potential sources originating from the abdominal wall are overlooked [8, 18]. This holds particularly true for TDH, which is a relatively uncommon phenomenon, yet it can manifest with intra-abdominal symptoms related to gynecological, urological, and gastroenterological processes [7–9, 19].

Carnett’s sign serves as a valuable clinical tool for early assessment during a patient’s examination, aimed at excluding a visceral source of pain and minimizing unnecessary investigations. This sign involves applying pressure to the most painful area of the patient’s abdomen while they are in a supine position, with their head and shoulders elevated from the examination table, thereby activating the abdominal muscles. If the pain remains unchanged or intensifies, Carnett’s sign is considered positive, suggesting a greater likelihood of an abdominal wall or extra-abdominal source of pain. Conversely, if the pain diminishes, the Carnett’s sign is considered negative, indicating a higher probability of a visceral source of pain, as the tensed abdominal muscles are presumed to provide protection to the intra-abdominal contents. [7].

While abdominal cutaneous nerve entrapment syndrome is typically not associated with abdominal wall bulges, it becomes an important consideration once an abdominal wall source has been identified. This syndrome is characterized by the entrapment of nerves at specific anatomical locations. It is noteworthy that muscle contraction during physical activity can exacerbate these entrapments, intensifying the associated pain. Among the various potential causes of abdominal wall pain, the most frequently encountered is the entrapment of the nerve at the lateral border of the rectus abdominus muscle. [18]. Ultrasound-guided injection of analgesic agents at the site of entrapment with subsequent pain relief confirms the diagnosis [20].

TDH manifesting as a flank bulge is an uncommon presentation that has only been described, to the best of our knowledge, in 7 other reports in the English literature (Table 1). There is no established standard treatment for cases of TDH accompanied by a pseudohernia and radicular pain. Within the existing literature, several approaches have been proposed to manage both the abdominal bulge and TDH. Among these, laminectomy in an option [3, 4]. Thoracic transforaminal epidural block [1], rehabilitation [13], NSAIDs alone [21], or pregabalin in combination with physiotherapy [13] have also been suggested as effective treatments.

**Table 1:** Summary of cases of thoracic disk herniation manifesting with abdominal pseudohernias

**Author and year**	**Age and sex**	**Level of herniation**	**Treatment**	**Treatment details**	**Pain improvement or complete resolution?**	**Bulging improved?**
Stetkarova I *et al.* 2007 [6]	50F	T12-L1	Conservative	Conservative treatment with Tramadol and NSAIDs for one month	Yes	NS
	34 M	T10-T12	Conservative	Periradicular treatment with injection of a mixture of methylprednisolone and 0.5% bupivacaine to the T12 root of the intervertebral foramen	Yes	NS
Myron M. LaBan *et al.* 2007 [2]	75 M	T12-L1	Surgical	Split-table intermittent pelvic traction after thermal therapy	Yes	NS
Zambelis T. *et al.* 2018 [22]	57 M	T11-T12	Conservative	Conservative treatment (not specified)	Yes	Yes, significantly subsided
Butenschoen V M *et al.* 2021 [3]	72F	T11-12	Surgical	Hemilaminectomy at T11 followed by bilateral sequestrectomy with semirigid fusion of T11–12	Yes	No, bulging stable at 5 months
Min Jong Ki *et al.* 2022 [1]	72F	T8-9-10-11	Conservative	Thoracic transforaminal epidural block	Yes	NS
Fitzpatrick, J. *et al.* 2022 [13]	57 M	T12-L1	Conservative	Regular clinical and EMG monitoring with rehabilitation.	Yes	Yes, complete resolution at 8 months
	67 M	T9-T10	Conservative	Pregabalin for 4 weeks and physiotherapy	Yes	Yes, 90% resolution at 5 months
Cho W. J. *et al.* 2023 [21]	35 M	T11-T12	Conservative	NSAIDs	Yes	Yes, complete resolution at 6 months
Present case	52 M	T11-T12	Conservative	NSAIDs, Pregabalin and muscle relaxants	Yes	Yes Complete resolution at 10 months
	57 M	T11-T12			Persistant paresthesia on T11-T12 dermatome	Yes Complete resolution at 10 months

Abbreviations: EMG: electromyography; NS: not specified; NSAID: nonsteroidal anti-inflammatory drug

The reasons for choosing between conservative and surgical treatment has not been consistently specified across articles. In one case, hemilaminectomy was performed due to acute denervation of the T11 root with persistent pain. Recurring symptoms at 8 weeks with abdominal wall bulging led to discectomy with T11-T12 fusion [3]. In another case, laminectomy was performed due to cord compression [4]. Among the case reports where conservative management was chosen, the main arguments were favorable clinical progression after initiation of medical treatment [13] and the absence of cord compression [6].

Although pain resolves with both conservative and surgical strategies, the evolution of abdominal wall bulging is inconsistently reported across articles. For instance, after surgery, Butenschoen *et al.* reported pain resolution but stability of the pseudohernia at 5-month follow-up [3]. In contrast, conservative management has been successful at resolving the bulging in four cases [13, 21, 22]. Of note, laminectomy is not always a permanent solution and could lead to acquired segmental denervation of abdominal wall muscles [14].

In this report, both patients were managed conservatively, using a combination of Pregabalin and muscle relaxants. Both presented considerable improvement on follow-up visits. Compared to the other conservative management suggested in the literature, the management option here aimed at minimizing opioid use.

Absolute indications for surgical management of any type of disk hernia include myelopathy or cauda equina syndrome, with deteriorating neurological deficits [23]. Furthermore, current guidelines recommend conservative management as a first line of treatment for classic TDH presenting with radicular pain. Surgery is recommended as second-line treatment, in the absence of absolute indications, if neurological symptoms worsen, or conservative management fails [24]. Most surgeons, however, suggest surgery as first-line treatment in cases of giant calcified hernia because they lead to myelopathy in 97% of cases [25]. Some also suggest surgery first, in cases of clear signs of myelopathy on MRI, before neurological symptoms appear or become irreversible [23]. In this report, conservative management was chosen for both patients, despite the atypical presentation of TDH due to the absence of absolute surgical indications.

Considering the evolution of both patients presented in this case report and the 3 patients treated conservatively in the literature, we estimate that a duration of 6–10 months is considered sufficient to reduce or resolve symptoms such as radicular pain and abdominal wall bulge. A transforaminal epidural block should be considered if symptoms persist for more than 12 months or continue to limit the patient’s daily activities at 10-month follow-up.

## Conclusion

Thoracic disk hernias are infrequent and often pose diagnostic challenges due to their diverse symptomatology. In this report, we presented two cases of TDH occurring at the T11-12 levels, which manifested with abdominal radicular pain accompanied by a left flank bulge that resembled an abdominal wall hernia. Both patients were managed conservatively, with minimal use of opioids. These findings emphasize the significance of considering spinal pathology in the differential diagnosis of flank bulges, even when abdominal hernia is initially suspected.
